# Cecal Diverticulitis: A Diagnostic Conundrum

**DOI:** 10.5811/westjem.2015.1.25119

**Published:** 2015-03-06

**Authors:** Kristof Nemeth, Sophie Vaughan

**Affiliations:** St. George’s Hospital, Department of Surgery, London, England, United Kingdom; Royal Gwent Hospital, Department of Radiology, Newport, Wales, United Kingdom

We report an unusual presentation of a 63-year-old female who presented with a five-day history of right-sided loin to groin pain. On assessment she was afebrile and her observations were stable. She had right iliac fossa pain and tenderness in the right renal angle. Urine dip was positive for blood only. A routine set of bloods revealed normal inflammatory markers and renal function. A differential diagnosis of renal colic was made as a cause for her symptoms, and after analgesia and intravenous (IV) fluids her symptoms settled and the patient was discharged with an urgent outpatient CT urogram the next day. She was readmitted two days later with recurrent symptoms and raised inflammatory markers. The CT from the previous day illustrated cecal diverticulitis (Figure 1 and Figure 2). The patient received 24 hours of IV antibiotics, and she was discharged on oral antibiotics the following day with a colonoscopy request to complete the assessment.

Cecal diverticulitis is a rare entity in Western countries. Its incidence is reported at 1–2% in Europe and the United States. 1 Right-sided diverticulae of the colon involve all layers of the colon making them true diverticulae, whereas left-sided diverticulae are false.

Clinical diagnosis is problematic, and the most common misdiagnosis is appendicitis resulting in surgical exploration. CT imaging has a sensitivity and specificity of 98% for appendicitis and has also the benefit of confirming or excluding alternative diagnoses such as renal colic.

CT has been shown to be cost effective in comparison to direct surgical exploration.^3^

The diagnosis of cecal diverticulitis is based on clinical judgment and radiological diagnosis. The management of the condition can be conservative if malignancy has been excluded or surgical. Surgical options include an isolated resection or when cecal adenocarcinoma cannot be excluded by a formal right hemicolectomy.^1^,^2^

## Figures and Tables

**Figure 1:**
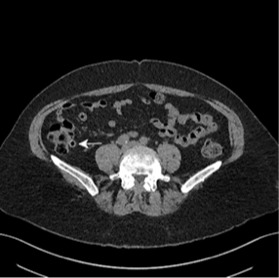
Axial view of the caecum with multiple diverticulae, one of which has a dense wall (white arrow).

**Figure 2:**
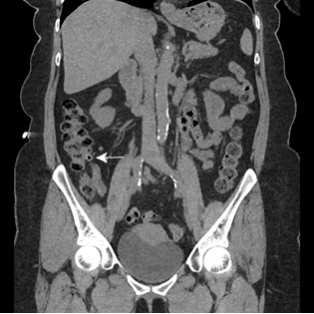
Coronal view of the caecum with multiple diverticulae, one of which has a dense wall (white arrow).
